# Influence of quinacrine and chloroquine on the in vitro 3′-azido-3′-deoxythymidine antiretroviral effect

**DOI:** 10.1186/s12981-015-0048-9

**Published:** 2015-03-19

**Authors:** Klintsy J Torres, Gustavo Reyes-Terán, Julio Sotelo, Helgi Jung-Cook, Lucinda Aguirre-Cruz

**Affiliations:** Laboratorio de Neuroimmunoendocrinología, Instituto Nacional de Neurología y Neurocirugía Manuel Velasco Suárez, Av. Insurgentes Sur 3877, 14269 México D F, México; Centro de Investigación en Enfermedades Infecciosas, Instituto Nacional de Enfermedades Respiratorias Ismael Cosío Villegas, Calzada de Tlalpan 4502, Col. Sección XVI, 14080 México D F, México; Laboratorio de Neuroinmunología y, ᅟ, ᅟ ᅟ, ᅟ; Laboratorio de Neuropsicofarmacología, Av. Insurgentes Sur 3877, Col. La Fama-Tlalpan, 14269 México D F, México; Departamento de Farmacia, Facultad de Química, Universidad Nacional Autónoma de México, Circuito Interior, Ciudad Universitaria, Col. Copilco, 04510 México D F, México

**Keywords:** Antimalarials, Quinacrine, Chloroquine, AZT, HIV

## Abstract

**Background:**

Antimalarials quinacrine (Qc) and chloroquine (Cq) intercalate DNA, potentiate the activity of other drugs and have lysosomotropic, anti-inflammatory and antiviral activities that could increase the effect of the 3′-azido-3′-deoxythymidine (AZT) antiretroviral agent. The aim of the current study was to evaluate if Qc and Cq could improve the in vitro effect of the antiretroviral AZT agent.

**Findings:**

Inhibition of viral replication in human immunodeficiency virus (HIV)_SF33_-infected peripheral blood mononuclear cells treated with Qc or Cq, alone or combined with a low dose of AZT was measured. Viral replication increased with Qc and decreased with high doses of Cq. The increase of replication caused by Qc was reversed by AZT. Neither Qc nor Cq significantly changed the antiviral activity of AZT.

**Conclusion:**

Cq does not potentiate the effect of AZT, but it is effective by itself at high doses. The rise of HIV replication by Qc could be deleterious in HIV endemic regions, where it is used as antimalarial. The mechanisms associated to this phenomenon must be identified.

## Findings

### Introduction

Human immunodeficiency virus (HIV)/acquired immunodeficiency syndrome morbidity and mortality have decreased with the use of antiretroviral therapy (ART). However, therapy adherence is frequently diminished by ART toxicity [1], which might in turn lead to emergence of resistant HIV strains [2].

Chloroquine (Cq) and quinacrine (Qc) have been widely used for decades as antimalarials, both have a well characterized biosafety and pharmacological profile [3]. The accumulation of Cq in breast milk of HIV infected women [4], and a decreased vertical transmission of HIV induced by this drug have been observed in malaria endemic countries [5]. Cq and Qc also have strong DNA intercalating properties [6], as well as special tropism for lysosomes [7], and concentrate for long periods in lymphoid tissue [8,9]. Due to the afore mentioned pharmacological properties, these drugs could be used as inhibitors of HIV replication [10,11]. Both drugs have also been linked to inhibition of: immune activation [12]; intracellular production of interferon [13]; nuclear factor-kappa B activation [14]; calcium signals in T cells [15]; and RNA polymerase activity [16]. Qc intercalation in viral DNA or RNA inhibits DNA mutations [17]. Additionally, Cq potentiates the activity of some antineoplastic drugs [18]. In this study, we evaluated the *in vitro* effect of Qc and Cq on HIV replication, administered alone or combined with low concentrations of the ART agent, 3′-azido-3′-deoxythymidine (AZT), in order to search for a possible additive effect of the antimalarial drug-AZT combinations.

## Methods

Peripheral blood mononuclear cells (PBMCs) provenient from different human voluntary healthy donors were used instead of CD4^+^ T cells, for better availability in our laboratory. The protocol was approved by the Research Board of the National Institute of Respiratory Diseases. PBMCs were purified by density gradient centrifugation and cultured at 37°C, 5% CO_2_ and 85% H_2_O, in RPMI-1640 medium, supplemented with fetal bovine serum (BioWhittaker, Anaheim CA), antibiotics Penicillin-Streptomicyn (BioWhittaker, Anaheim CA), L-Glutamine (BioWhittaker, Anaheim CA) and human IL-2 (20 U/ml, Roche, USA), as previously described [19]. In order to determine the cytotoxicity effect, the viability of uninfected PBMCs, treated at the same drug concentrations, used in the drug assay, was measured using trypan blue exclusion staining (0.04%, Cambrex, USA). The percentage of viable cells was determined dividing the number of alive cells × 100/total cells. HIV_SF33_ virus isolated and donated by Levy et al. [20], was tittered on phytohemagglutinin-stimulated PBMCs for determining the tissue culture infectious dose 50 (TCID_50_), as described by McDougal [21]. Viral stocks were then stored frozen at −80°C until their use. PMBCs were infected with HIV_SF33_ (500 TCID_50_/1×10^6^ cells) by 2 h at 37°C, and extensively washed to remove the virus, and cultured during 4 days in the presence of the following treatments: Phosphate Buffered Saline (PBS, 15 mM as control); AZT (0.008 μM, ID_50_); Qc (0.4-2 μM); Cq (5–20 μM); or the mixtures Qc (0.4-2 μM) + AZT (0.008 μM); Cq (5–20 μM) + AZT (0.008 μM). On the fourth day of culture, the viral replication, measured as level of HIV-1 p24 antigen, was tested by Enzyme-Linked ImmunoSorbent Assay (ELISA, Beckman Coulter, Fullterton, CA). Absorbance (450 nm) was measured with the CODA EIA automated Analyzer (Bio-Rad, Hercules, CA) and concentration was calculated with CODA software. All assays were run in triplicate on three different days (n = 9), using the same HIV_SF33_ virus stock. The percentage of inhibition of replication was determined by calculating percent reduction HIV p24 antigen in wells containing the drugs and were compared with control (PBS) using the formula: HIV p24 antigen in PBS - HIV p24 antigen drug treatment × 100/HIV p24 antigen in PBS. Statistical analysis included Student’s *t*-test to compare cell viability between drug treatment alone or combined and one-way analysis of variance (ANOVA) test followed by the Tukey’s post-hoc test to compare inhibition of HIV replication between AZT and Cq or Qc treatments using the GraphPad Prism 6 software. Differences were considered significant when p-value was < 0.05.

## Results

Table 1 shows the cell viability in cultures treated with AZT, Qc or Cq. It can be seen that the tested dose of AZT (0.008 μM) did not reduce cell viability compared to PBS control PBMCs. Both Qc and Cq produced dose-related decreases in cell viability, with significant reductions in percentage occurring at the highest concentrations of Qc (5 μM, p = 0.038) and Cq (20 μM, p = 0.004), when compared with PBS control. Addition of AZT to Qc or Cq did not cause any further increase in cytotoxicity.

**Table 1:** **Viability of peripheral blood mononuclear cells treated with PBS, quinacrine, chloroquine or 3′-azido-3′-deoxythymidine** ^**a**^

**Drug**	**Dose (μM)**	**Treatment**		**Treatment combined with AZT (0.008 μM)**		
		**Cells** ^**b**^ **(mean ± SD)**	**% of viability**	**Cells** ^**b**^ **(mean ± SD)**	**% of viability**	**(p)** ^**c**^
PBS (Control)	-	247 ± 61	87 ± 7	-	-	-
3′-azido-3′-deoxythymidine, AZT	0.008	238 ± 70	85 ± 6	-	-	-
Quinacrine, Qc	0.4	213 ± 66	88 ± 8	198 ± 63	90 ± 5	ns
	1	186 ± 96	83 ± 14	208 ± 60	86 ± 7	ns
	5	139 ± 69	71 ± 17^d^	126 ± 59	71 ± 12	ns
Chloroquine, Cq	5	216 ± 64	88 ± 8	210 ± 83	89 ± 5	ns
	10	184 ± 67	86 ± 10	179 ± 60	84 ± 7	ns
	20	106 ± 36	68 ± 17^e^	99 ± 47	63 ± 10	ns

^a^Purified uninfected PBMCs were treated with PBS, Qc, Cq or AZT and incubated during 4 days. Cell viability was determined by using trypan blue exclusion staining. Dead (blue) and alive (unstained) cells were counted. The percentage of viable cells was determined dividing the number of alive cells × 100/total cells.

^b^Mean value (+/− standard deviation) of viable cells from three independent assays by triplicate (n = 9).

^c^Student’s *t*-test between treatment alone and combined with AZT at the same concentration.

^d^Post-hoc Tukey’s test of concentration compared with control, p = 0.0363.

^e^Post-hoc Tukey’s test of concentration compared with control, p = 0.0033.

ns = not significant.

**Figure 1:**
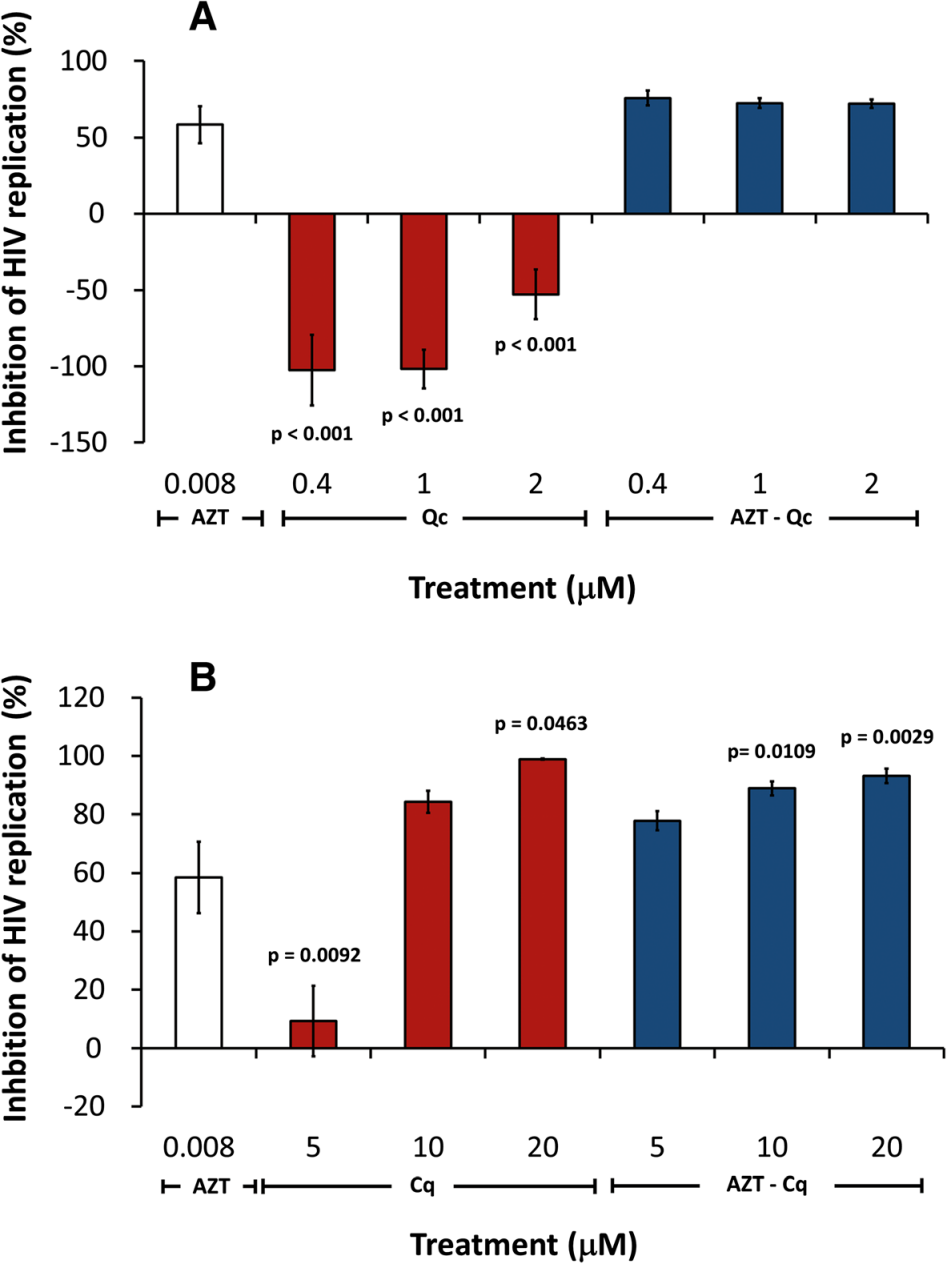
**In vitro inhibition of HIV replication by different drug treatments: Quinacrine (A) and Chloroquine (B).** HIV p24 antigen (ng/ml) was measured in supernatants of cultures of peripheral blood mononuclear cells, infected with the HIV_SF33_ strain. Cells were treated during 4 days with 3′-azido-3′-deoxythymidine (AZT) (control), Quinacrine (Qc), Chloroquine (Cq) or the mixtures Qc-AZT or Cq-AZT. Each bar represents the mean +/− standard error of three independent assays by triplicate (n = 9). One-way ANOVA, followed by Tukey’s post-hoc test, was used to compare: **A**, Control (AZT) and Qc or AZT- Qc; and **B**, Control (AZT) and Cq or AZT-Cq. Statistical significant p-values are shown in the figure.

The low dose of AZT used in this report was selected to yield modest inhibition of HIV_SF33_ replication so that effects of coadministration of Qc or Cq could be observed. Treatment with 0.008 μM AZT alone significantly inhibited HIV_SF33_ replication by nearly 50% (*t*-test, p = 0.0061) compared to PBS. Qc (0.4-2 μM) administered alone caused increases in HIV replication that appeared to be inversely related to dose of the antimalarial drug (Figure 1A) (p = <0.0001). This enhancement of viral replication by Qc alone was reversed by co-administration of AZT, with the level of inhibition of viral replication produced by Qc plus AZT being similar to that provided by AZT alone (Figure 1A). In contrast, administration of Cq alone at low dose (5 μM) yielded modest, but significant inhibition of viral replication of 10%, while higher dose of Cq (20 μM) inhibited HIV replication around 80% (Figure 1B) (p = 0.0463). Inhibition of replication by AZT-Cq combination was higher to that obtained with AZT alone (p = 0.0035), but was similar to that obtained with Cq alone (10 μM) (Figure 1B).

## Discussion

The aim of the current study, was to evaluate if Qc and Cq could improve the in vitro effect of the antiretroviral AZT agent. Qc and Cq were evaluated at different concentrations, administered alone or combined with AZT.

AZT is one of the drugs used in the ART in developing countries of low incomes, where many people do not have access to the expensive new antiretroviral agents [22]. Cq, a widely used drug for the treatment of malaria, has shown antiviral effects [10], lysosomotropic activity [7] and a preferential affinity for lymphoid tissues [8,9], useful properties against HIV. In turn, the anti-HIV effect of Qc, other DNA-intercalating agent [6], has not been evaluated.

Results showed that the treatment of HIV-infected PBMCs with Qc alone, increased HIV replication. This result had not been reported before, but some studies have shown a similar effect with cocaine, explained by a possible regulation of the HIV entry in the cells and anti-HIV microRNAs [23,24]. When the combination Qc-AZT was evaluated, the HIV inhibition was similar to that obtained with AZT alone, which could be explained by the anti-HIV effect of AZT.

Previous reports have shown that Cq inhibits viral replication [25]. In the present study, the same effect was observed at high doses (10–20 μM). When these doses were combined with AZT, the inhibition was similar than that obtained with Cq alone, and higher than AZT. This result suggests that the HIV inhibition is related to the intrinsic effect of Cq. The inhibition of HIV replication with a low dose of Cq-AZT (5 and 0.008 μM, respectively) was not significantly different from that observed by administration of AZT alone. Considering that both effects are independent, the combination AZT-Cq does not improve the anti-HIV effect.

## Conclusion

Given our results, the use of Qc as antimalarial agent in untreated HIV patients should be carefully observed, due to the possible increase of HIV viral load. We can conclude that, neither Cq or Qc improve the efficacy of AZT.

## Abbreviations

ART: Antiretroviral therapy
AZT: 3′-azido-3′-deoxythymidine
Cq: Chloroquine
ELISA: Enzyme-Linked Immunosorbent Assay
HIV: Human Immunodeficiency Virus
ID_50_: Inhibitory Dose 50%
NS: Not significant
PBMCs: Peripheral blood mononuclear cells
PBS: Phosphate buffered saline
Qc: Quinacrine
TCID_50_: Tissue culture infectious dose 50%
